# Chitosan Wound Dressings Incorporating Exosomes Derived from MicroRNA‐126‐Overexpressing Synovium Mesenchymal Stem Cells Provide Sustained Release of Exosomes and Heal Full‐Thickness Skin Defects in a Diabetic Rat Model

**DOI:** 10.5966/sctm.2016-0275

**Published:** 2016-10-26

**Authors:** Shi‐Cong Tao, Shang‐Chun Guo, Min Li, Qin‐Fei Ke, Ya‐Ping Guo, Chang‐Qing Zhang

**Affiliations:** ^1^ Department of Orthopedic Surgery, Shanghai Jiao Tong University Affiliated Sixth People’s Hospital, Shanghai, People’s Republic of China; ^2^ Institute of Microsurgery on Extremities, Shanghai Jiao Tong University Affiliated Sixth People’s Hospital, Shanghai, People’s Republic of China; ^3^ The Education Ministry Key Lab of Resource Chemistry and Shanghai Key Laboratory of Rare Earth Functional Materials, Shanghai Normal University, Shanghai, People’s Republic of China

**Keywords:** Exosomes, Control release, Wound healing, Angiogenesis, Synovium mesenchymal stem cells, Gene modification

## Abstract

There is a need to find better strategies to promote wound healing, especially of chronic wounds, which remain a challenge. We found that synovium mesenchymal stem cells (SMSCs) have the ability to strongly promote cell proliferation of fibroblasts; however, they are ineffective at promoting angiogenesis. Using gene overexpression technology, we overexpressed microRNA‐126‐3p (miR‐126‐3p) and transferred the angiogenic ability of endothelial progenitor cells to SMSCs, promoting angiogenesis. We tested a therapeutic strategy involving controlled‐release exosomes derived from miR‐126‐3p‐overexpressing SMSCs combined with chitosan. Our in vitro results showed that exosomes derived from miR‐126‐3p‐overexpressing SMSCs (SMSC‐126‐Exos) stimulated the proliferation of human dermal fibroblasts and human dermal microvascular endothelial cells (HMEC‐1) in a dose‐dependent manner. Furthermore, SMSC‐126‐Exos also promoted migration and tube formation of HMEC‐1. Testing this system in a diabetic rat model, we found that this approach resulted in accelerated re‐epithelialization, activated angiogenesis, and promotion of collagen maturity in vivo. These data provide the first evidence of the potential of SMSC‐126‐Exos in treating cutaneous wounds and indicate that modifying the cells—for example, by gene overexpression—and using the exosomes derived from these modified cells provides a potential drug delivery system and could have infinite possibilities for future therapy. Stem Cells Translational Medicine *2017;6:736–747*

**Significance Statement**

There is a need to find better strategies to promote wound healing, especially of chronic wounds, which remain a challenge. Synovium mesenchymal stem cells (SMSCs) were found to have the ability to strongly promote cell proliferation of fibroblasts; however, they are ineffective at promoting angiogenesis. Gene overexpression technology was used to overexpress microRNA‐126‐3p (miR‐126‐3p) and transfer the angiogenic ability of endothelial progenitor cells to SMSCs, promoting angiogenesis. This research provides the first evidence of the potential of exosomes derived from miR‐126‐3p‐overexpressing SMSCs in treating cutaneous wounds and indicates that modifying the cells—for example, by gene overexpression—and using the exosomes derived from these modified cells provides a potential drug delivery system and could have infinite possibilities for future therapy.

## Introduction

Diabetes mellitus is a rapidly increasing health problem with the world prevalence of diabetes affecting those between 20 and 79 years of age expected to reach 7.7% (439 million adults) by 2030 [1]. Chronic foot ulcers are one of the most debilitating complications of diabetes mellitus [2]. In chronic ulcers, the healing process is prolonged because of poor perfusion and the presence of necrotic tissue [3]. The regeneration of tissue with appropriate angiogenesis is essential for healing of chronic wounds. Blood vessels supply soluble factors and circulating stem or progenitor cells to sites of tissue regeneration, as well as provide nutrients to cells and removing waste products [4].

MicroRNAs (miRNAs) are noncoding RNAs composed of approximately 22 nucleotides that target mRNAs for translational repression or cleavage before the regulation of gene expression [5]. miRNA‐126 (miR‐126) is one of the miRNAs that plays proangiogenic roles in the vasculature of both endothelial cells and perivascular cells [6]. By enhancing the proangiogenic actions of fibroblast growth factor and vascular endothelial growth factor, miR‐126 promotes blood vessel formation [7, 8, 9, 10]. miR‐126 has also been proven essential for the angiogenic ability of endothelial progenitor cells (EPCs) [11, 12], which are involved in neoangiogenesis and repair in ischemic diseases [13]. Transplantation of mesenchymal stem cells (MSCs) transfected with miR‐126 has been shown to improve angiogenesis in the repair of ischemic diseases [14] through the phosphatidylinositol 3‐kinase (PI3K)/AKT and mitogen‐activated protein kinase (MAPK)/extracellular signal‐regulated kinase (ERK) pathways [15, 16].

MSCs derived from the synovial membrane, named synovium mesenchymal stem cells (SMSCs), were first isolated in 2001 [17]. The most attractive aspect of SMSCs is their tissue‐specific nature, which is supported by increased cell proliferation of connective tissue without promotion of adipogenesis or osteogenesis [18, 19], and this high tissue specificity leads to highly efficient repair. Because of these properties, we created a cell line of advanced seed cells overexpressing miR‐126‐3p, for use in tissue engineering of wound healing, which has better angiogenic activity than its sister miR‐126‐5p [10, 20] in SMSCs.

However, direct cell transplantation of stem cells is still associated with problems including potential immunological rejection and neoplasm formation [21, 22]. Consequently, it is necessary to enhance the advantages of stem cells and minimize the disadvantages of cell transplantation.

In the 1980s, Rose Johnstone first described exosomes as nanoscale membrane vesicles (30–150 nm in diameter) [23]. They can be identified by their expression of exosome‐associated markers such as Alix, Tsg101, CD9, CD63, and CD81 [24, 25, 26] and carry a complex cargo that include proteins, lipids, and nucleic acids—of which miRNAs are functional complexes that are implicated in tissue repair and regeneration [26]. Thus, exosomes are considered as drug‐delivery vehicles for treatment of diseases [27, 28] and naturally occurring RNA carriers, which can even deliver therapeutic short interfering RNA to target cells [29].

Recent research has shown that activation of resident cells via a paracrine mechanism may play a leading role in stem cell‐mediated or progenitor cell‐mediated tissue regeneration [30, 31]. Exosomes are considered to be one of the most important secretory products of MSCs, mediating intercellular communication and enhancing wound healing [32].

It is well known that chitosan (CS) hydrogel is a hemostatic, antibacterial, biodegradable, and biocompatible carrier for sustained release of nanoparticles [33, 34]. The aim of our study was to develop and apply low‐temperature polymerized CS to reduce the damage and prolong the delivery of exosomes derived from miR‐126‐3p‐overexpressing SMSCs to diabetic wounds.

## Materials and Methods

### Isolation and Characterization of SMSCs

Biopsies of synovial membrane (wet weight 20–50 mg) were obtained aseptically during arthroscopically assisted surgery. Synovial membrane specimens were rinsed twice with phosphate‐buffered saline solution (PBS) supplemented with penicillin‐streptomycin (PS; 100 units/ml penicillin, 100 μg/ml streptomycin; Thermo Fisher Scientific Life Sciences, Oakwood Village, OH, https://www.thermofisher.com), minced, and digested with 0.2% collagenase, type I (Thermo Fisher) in high‐glucose Dulbecco’s modified Eagle’s medium (high‐glucose DMEM; Thermo Fisher) containing 10% fetal bovine serum (FBS; Thermo Fisher). After incubation at 37°C overnight, cells were collected by centrifugation, washed twice, and resuspended in high‐glucose DMEM supplemented with 10% FBS and PS. Resuspended cells were planted into a T25 culture flask and then incubated for 4 days to allow cells to attach. After changing the medium to remove nonadherent cells, the medium was replaced every 3 days. Cells were cultured in monolayer in high‐glucose DMEM supplemented with 10% FBS and PS at 37°C in a humidified atmosphere of 5% CO_2_.

Surface antigens of SMSCs were analyzed by flow cytometry. Cells at passage 5 were harvested by using trypsin‐EDTA (0.25%; Thermo Fisher) and incubated with 3% bovine serum albumin (BSA; Thermo Fisher) in PBS for 30 minutes to block nonspecific antigen binding before incubating with the following antibodies (all from BD, Franklin Lakes, NJ, http://www.bd.com): allophycocyanin (APC)‐conjugated anti‐CD34, phycoerythrin (PE)‐conjugated anti‐CD44, PE‐conjugated anti‐CD73, and fluorescein isothiocyanate (FITC)‐conjugated anti‐CD45. The treated cells were analyzed by using a Guava easyCyte flow cytometer (Merck Millipore, Darmstadt, Germany, http://www.emdgroup.com). The capacity of SMSCs to differentiate into osteogenic, adipogenic, and chondrogenic lineages was tested by using specific differentiation medium.

### Lentivirus Transfection

The miR‐126‐3p lentiviral vector was obtained from GenePharma (Shanghai, People’s Republic of China, http://www.genepharma.com/En). Lentiviral transfection was performed according to the manufacturer’s protocol. Briefly, SMSCs were incubated in retroviral supernatant with 5 μg/ml polybrene for 24 hours. After infection for 48 hours, SMSCs were selected with puromycin dihydrochloride (Thermo Fisher).

### Isolation and Identification of Exosomes

After SMSCs reached approximately 50%–60% confluence, the supernatant was discarded, and the cells were washed with PBS, then MesenGro hMSC medium (StemRD, Burlingame, CA, http://www.stemrd.com) was added, and the cells were cultured for 48 hours. The conditioned medium (CM) from SMSCs was collected, and exosomes were isolated as previously described [35, 36, 37, 38]. Briefly, the CM was centrifuged at 300*g* for 15 minutes, followed by 2,000*g* for 15 minutes to remove dead cells and cellular debris. Then the supernatants were filtered through a 0.22 μm filter (Merck Millipore) to further remove cellular debris in the CM. The filtered solution was centrifuged at 4,000*g* until the volume in the upper compartment was concentrated to approximately 200 μl in a 15‐ml Amicon Ultra‐15 Centrifugal Filter Unit (Merck Millipore). The ultrafiltration liquid was washed with PBS, and ultrafiltration was repeated three times at 4,000*g* to 200 μl. For further purification, the liquid was ultracentrifuged at 100,000*g* for 1 hour using a sterile Ultra‐Clear tube (Beckman Coulter, Brea, CA, https://www.beckmancoulter.com) with a 30% sucrose‐D_2_O cushion. The pellets were resuspended in 15 ml of PBS and centrifuged at 4,000*g* to approximately 200 μl. All procedures were performed at 4°C.

Exosome morphologies were observed using transmission electron microscopy (TEM). The size distribution of exosomes was measured by using Nanosizer technology (Malvern Instruments, Malvern, U.K., http://www.malvern.com). Expression of the exosomal characteristic markers Alix (1:500), CD9 (1:500), CD63 (1:1,000), CD81 (1:1,000), and TSG101 (1:500) (all from System Biosciences, Palo Alto, CA, https://www.systembio.com) were analyzed by Western blotting.

### Preparation of Chitosan Hydrogel Loaded With Exosomes

CS (0.12 g; Sinopharm Chemical Reagent Co., Ltd, Shanghai, People’s Republic of China, http://en.reagent.com.cn) was dissolved in 3.6 ml of acetic acid solution (2.0 vol%) by stirring for 2 hours to form a homogeneous CS solution. Then, 1.2 ml of exosome solution (50wt%) was added to the CS solution, followed by stirring for 5 hours. Aliquots of 0.5 ml of the above mixture were transferred into a 24‐well plate and placed at −20°C for 2 hours, and then 5.0wt% NaOH solution was added and the mixture was kept at 4°C for 4 hours. Finally, the product, exosomes derived from miR‐126‐3p‐overexpressing SMSCs treated with CS (CS‐SMSC‐126‐Exos), was washed with deionized water. Control CS hydrogel was prepared under the same conditions, with the addition of PBS alone instead of exosomes.

### Characterization of Materials

In order to characterize the phase, structure, morphology, and thermal properties of CS‐SMSC‐126‐Exos and CS hydrogel, the samples were freeze‐dried and then analyzed by x‐ray diffraction (XRD) (D/Max‐II B, Rigaku, Tokyo, Japan, http://www.rigaku.com/en) with CuKa radiation (λ = 1.541874 Å) to characterize the phase structure within the scanning range of 2Ө = 5–80°. Morphological images of the samples were acquired on a Hitachi S‐4800 scanning electron microscope (SEM; Hitachi, Tokyo, Japan, http://www.hitachi.com), and the corresponding element compositions were detected by energy‐dispersive spectroscopy (EDS). The functional groups of CS and exosomes were detected by Fourier transform IR (FTIR) spectrometry (Vector22; Bruker Daltonics, Billerica, MA, https://www.bruker.com) using the KBr pellet technique. The thermal behaviors of samples were characterized by thermo‐gravimetric analysis (TG‐DTA; PerkinElmer, Waltham, MA, http://www.perkinelmer.com) in an air atmosphere.

### Concentration Time Curve

The materials loaded with exosomes were immersed in MesenGro hMSC medium (System Biosciences) for 3, 6, or 12 hours or 1, 2, 3, 4, 5, or 6 days. Material after 6‐day immersion was also dissolved for measuring residue of exosome after 6‐day immersion to calculate the total load of exosome. The particle number of exosomes was determined by using a CD63 ExoELISA kit (System Biosciences) following the instructions provided. Briefly, a standard curve was prepared by serially diluting the ExoELISA protein standard with exosome binding buffer, and results were quantitated by using a microplate reader (Bio‐Rad, Hercules, CA, http://www.bio‐rad.com).

### Isolation of miRNA From Cells and Exosomes

Total RNA was extracted from cells by using TRIzol Reagent and from exosomes by using a Total Exosome RNA & Protein Isolation Kit (both from Thermo Fisher) following the manufacturer’s instructions. Synthesis of cDNA and quantitative reverse‐transcriptase polymerase chain reaction (qRT‐PCR) was performed by using a human microRNA qRT‐PCR detection kit specifically for hsa‐miR‐126 and RNU6B (BioTNT, Shanghai, People’s Republic of China, http://www.biotnt.com). qRT‐PCR was performed with an ABI 7900HT System (Applied Biosystems, Foster City, CA, http://www.appliedbiosystems.com) using the primers listed below. RNU6B was used for normalization of the results.

Primers were as follows: reverse transcription primer for mir‐126‐3p, CTCAACTGGTGTCGTGGAGTCGGCAATTCAGTTGAGCGCATTAT;forward primer for mir‐126‐3p, CGTACCGTGAGTAATAATG; reverse primer for mir‐126‐3p, AACTGGTGTCGTGGAG; reverse transcription primer for RNU6B, CTCAACTGGTGTCGTGGAGTCGGCAATTCAGTTGAGAAAAATAT; forward primer for RNU6B, CAAGGATGACACGCAAAT; reverse primer for RNU6B, TGGTGTCGTGGAGTCG.

### In Vitro Response of Cells to SMSC‐Exos and SMSC‐126‐Exos

#### Cells and Cell Culture

The response of cells to the exosomes was studied in vitro. Sample extraction, a widely used method of evaluating materials whose functions are performed by released components [39, 40], was used in this study. Specifically, the cells were incubated in one of two representative concentrations of exosomes released from the material after immersion for 24 hours or 6 days. Proliferation, migration, tube formation, and signal pathways were then analyzed.

Human dermal microvascular endothelial cells (HMEC‐1 cells) were cultured in MCDB131 medium (Thermo Fisher) supplemented with 10% FBS (Thermo Fisher), 2 mM L‐glutamine, 10 ng/ml epidermal growth factor (Sigma‐Aldrich, St. Louis, MO, http://www.sigmaaldrich.com) and 1 μg/ml hydrocortisone (Sigma‐Aldrich). The human fibroblasts (FBs) used in the present study were approved by the ethical committee of Shanghai Sixth People’s Hospital, Shanghai Jiaotong University School of Medicine. This study was in adherence with the Declaration of Helsinki. Written informed consent was obtained from each human volunteer.

Primary dermal FBs were isolated and cultured using a previously described method [41]. Briefly, skin specimens from donors were cut into small pieces and placed in a 15‐cm culture dish before being cultured in high‐glucose DMEM supplemented with 10% FBS and PS.

#### Cell Proliferation

The effect of the exosomes on proliferation of HMEC‐1 cells and FBs was measured by using a Cell Counting Kit‐8 (CCK‐8; Dojindo Molecular Technologies, Inc., Kumamoto, Japan, http://www.dojindo.com) according to the manufacturer’s instructions. Briefly, HMEC‐1 cells or FBs were seeded into 96‐well plates at an initial density of 5 × 10^3^ cells per well and cultured in different sources or concentrations of exosomes for 1, 3, or 5 days. The medium was changed every day and replaced with fresh medium containing the same source of exosomes. Then, 180 μl of culture medium mixed with 20 μl of CCK‐8 were added into each well at the appropriate time point and incubated for 1 hour at 37°C before measuring product concentration with a microplate reader at a wavelength of 450 nm.

#### HMEC‐1 Migration and Tube Formation

The effect of exosomes on HMEC‐1 migration was evaluated by using a transwell assay [42]. Briefly, 5 × 10^4^ cells were seeded into the upper chamber of a 24‐well transwell plate (Corning, Corning, NY, http://www.corning.com; pore size = 8 μm). Then, 600 μl of medium containing different types and concentrations of exosomes was added into the lower chamber. After incubation for 6 hours, cells were fixed with 4% paraformaldehyde and then stained for 5 minutes with 0.5% crystal violet. The cells on the upper surface of the membranes were removed with a cotton swab after washing three times in PBS. Four randomly selected fields (×100) per filter were evaluated twice by two independent evaluators in a blinded manner.

In vitro capillary‐network formation in Matrigel matrix (BD Biosciences, San Jose, CA, http://www.bdbiosciences.com) was monitored by evaluating the effects of exosomes on tube formation activity of HMEC‐1 cells.

### Rat Skin Wound Model and Treatment

All experimental protocols were approved by the Animal Research Committee of Shanghai Jiaotong University School of Medicine. Adult male Sprague‐Dawley rats weighing 300–350 g were used in this study. These rats were given an intraperitoneal (i.p.) injection of streptozotocin [43] (Sigma‐Aldrich) at a dose of 55 mg/kg in 0.05 mol/l sodium citrate buffer, pH 4.5. Blood glucose level was monitored every 3 days by using an OneTouch UltraEasy monitoring system (LifeScan, Milpitas, CA, https://www.lifescan.com) from tail vein blood. A prolonged diabetic status was defined as a glucose level no lower than 20 mmol/l during the induction period. Animals were anesthetized by i.p. injection of 50 mg/kg pentobarbital, and defect areas were marked by using a ring‐shaped seal (18 mm in diameter). Standardized full‐thickness skin wounds were created by excising the dorsal skin under aseptic conditions. Test materials were placed on the wound bed, and each rat received a pressure dressing (Tegaderm film, 3M, St Paul, MN, http://www.3m.com) to secure the materials. After surgery, the rats were observed every day to ensure that the materials and pressure dressing remained intact. The wounds were photographed on days 0, 3, 7, and 14 after surgery by using a D810 camera (Nikon, Tokyo, Japan, http://www.nikon.com). The wound area was measured using ImageJ software [44] (NIH, Bethesda, MD, https://imagej.nih.gov/ij). Wound‐size reduction was determined by using the following formula: wound‐size reduction (%) = (*A*_0_ − *A*_t_)/*A*_0_ × 100, where *A*_0_ is the initial wound area (at *t* = 0) and *A*_t_ is the wound area at day 3, 7, or 14 after surgery.

All animal experiments complied with the Animal Research: Reporting of In Vivo Experiments guidelines. All experimental protocols were approved by the Animal Research Committee of Shanghai Jiao Tong University School of Medicine.

### Histology, Immunofluorescence, and Immunochemical Analysis

Six wounds per group were studied by histopathological analysis on days 7 and 14 after surgery. The tissue comprising the wound bed and surrounding healthy skin was removed and fixed in 4% paraformaldehyde overnight. The excised skin was then dehydrated through a graded series of ethanol, embedded in paraffin, and sectioned perpendicularly to the wound surface into 5‐μm‐thick sections. The sections were stained by using hematoxylin and eosin (H&E) for histological observation. Masson’s trichrome was used to determine the degree of collagen maturity. The length of the neoepithelium was determined by using a previously described procedure [41].

Immunofluorescence (IF) and immunohistochemical (IHC) staining of the sections were performed to reveal the progress of angiogenesis during wound healing. Immunofluorescent staining of CD31, a marker of endothelial cells (1:50; Abcam, Cambridge, U.K., http://www.abcam.com) and α‐smooth muscle actin (α‐SMA), a marker of vascular smooth muscle (1:100; Abcam), and IHC staining for CD31 were performed to reveal the regenerated arterioles and capillaries.

For immunofluorescent staining, tissue sections were rehydrated, blocked in 1.5% goat serum (Merck Millipore) for 60 minutes at room temperature (RT), incubated in the primary antibody overnight at 4°C, and then stained with FITC‐ or Cy3‐conjugated secondary antibodies and counterstained with 4′,6‐diamidino‐2‐phenylindole. Images were acquired with an LSM‐880 confocal‐microscope (Carl Zeiss Microscopy GmbH, Jena, Germany, http://www.zeiss.com).

### Microfil Perfusion and Microcomputed Tomography

To evaluate new blood vessel formation, after the rats were euthanized at 14 days after surgery, they were perfused with Microfil (Microfil MV‐122; Flow Tech, Carver, MA, http://www.flowtech‐inc.com) by using a previously described method [40]. The samples were allowed to stand overnight at 4°C to ensure sufficient polymerization, and the next day they were analyzed by microcomputed tomography (μCT) (Skyscan 1176; Bruker) at a resolution of 9 μm to detect new blood vessels. Three‐dimensional images were reconstructed by using the CTVol program (Bruker). The area and number of blood vessels in the defect were also determined by using this software together with ImageJ.

### Statistical Analysis

All data are expressed as mean ± SD. One‐way analysis of variance was used to determine the level of significance using GraphPad Prism software, and *p* values < .05 were considered statistically significant.

## Results

### Characterization of SMSCs and SMSC‐Exos

**Figure 1:**
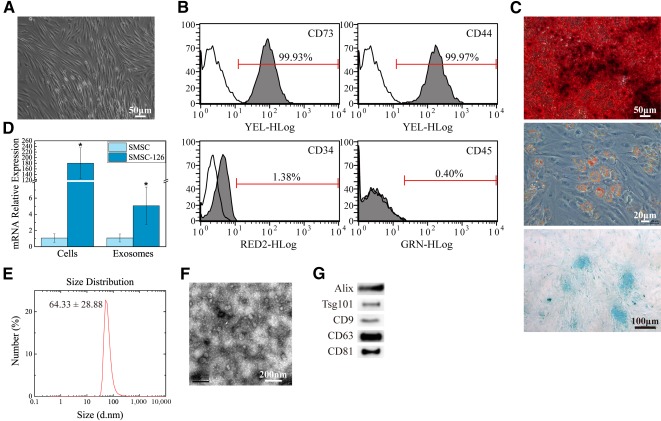
Classification and modification of human SMSCs and exosomes. **(A):** SMSCs exhibited a spindle‐like morphology. Scale bar = 50 μm. **(B):** Flow cytometric analysis of cell surface markers. Blank curves represent isotype controls and solid gray curves represent test samples. **(C):** SMSCs displayed osteogenic, adipogenic, and chondrogenic differentiation potential. Scale bars = 50 μm (osteogenic), 20 μm (adipogenic), and 100 μm (chondrogenic). **(D):** mRNA levels detected by quantitative reverse‐transcriptase polymerase chain reaction. The experiment was repeated three times. ∗, *p* < .05 compared with unmodified SMSCs. **(E):** Particle size distribution of SMSC‐Exos measured by Nanosizer. The experiments were repeated three times, and representative results are shown. **(F):** Morphology of SMSC‐Exos analyzed by transmission electron microscopy. Scale bar = 200 nm. **(G):** Exosome surface markers detected by Western blotting (Alix, Tsg101, CD9, CD63, and CD81). Three independent experiments were performed to confirm the stability of these phenomena. Abbreviations: SMSC, synovium mesenchymal stem cell; SMSC‐126, microRNA‐126‐3p‐overexpressing synovium mesenchymal stem cells.

SMSCs were successfully isolated as described in Materials and Methods: Isolation and Characterization of SMSCs. After the first passage and throughout expansion, the population of SMSCs appeared to be relatively homogeneous, composed of spindle‐like cells (Fig. 1A). The identification of SMSCs was confirmed by staining with surface markers, followed by flow‐cytometric analysis (Fig. 1B). SMSCs were identified as cells positive for the mesenchymal stem cell marker CD73 and the synovium‐derived MSC markers CD44 and negative for CD34 and CD45. Trilineage differentiation experiments of SMSCs were performed to assess their pluripotency (Fig. 1C). Their osteogenic differentiation potential was studied by measuring the formation of calcium mineral deposits by Alizarin Red staining after 2 weeks of differentiation. Chondrogenic differentiation potential was studied by determining the presence of polysaccharides and proteoglycans by Alcian Blue staining after 4 weeks of differentiation, whereas adipogenic differentiation potential was evaluated by measuring the formation of small cytoplasmic lipid droplets, revealed by Oil Red O staining after 2 weeks of differentiation. These results confirmed that the SMSCs possessed MSC properties and pluripotency.

The size of SMSC‐derived exosomes (SMSC‐Exos) was directly measured by using the Nanosizer system (Fig. 1E). In TEM experiments with SMSC‐Exos, we observed spherical microvesicles that were 30–150 nm in diameter (Fig. 1F), indicating the presence of exosomes. Western blotting showed that the SMSC‐Exos expressed the exosomal markers Alix, Tsg101, CD9, CD63, and CD81 (Fig. 1G).

### Structure, Morphology, and Thermal Behavior of CS‐SMSC‐126‐Exos

**Figure 2:**
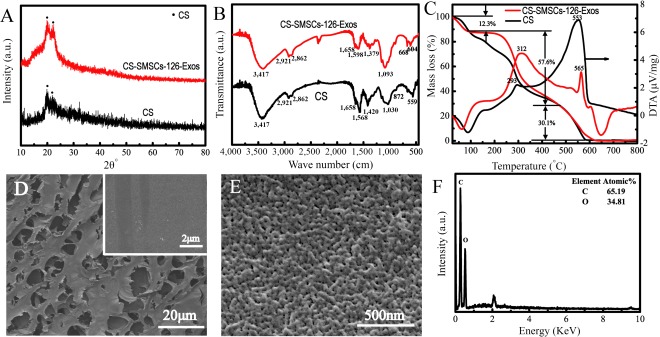
Characterization of materials. **(A):** X‐ray diffraction patterns. **(B, C):** Fourier transform IR spectra **(B)** and thermo‐gravimetric analysis curves **(C)** of CS‐SMSC‐126‐Exos and CS hydrogel. **(D–F):** Scanning electron microscopy images **(D, E)** and energy‐dispersive spectroscopy spectrum **(F)** of CS‐SMSC‐126‐Exos. Abbreviations: a.u., arbitrary units; CS, chitosan; CS‐SMSC‐126‐Exos, exosomes derived from microRNA‐126‐3p‐overexpressing synovium mesenchymal stem cells combined with chitosan; deg., degrees; DTA, thermo‐gravimetric analysis.

Freeze‐drying was used to dry the CS‐SMSC‐126‐Exos and CS hydrogel because they contain a large amount of water. The phase, functional groups, and morphology and thermal behavior of CS‐SMSC‐126‐Exos and CS hydrogel were characterized from their XRD patterns, FTIR spectra, TG‐DTA curves, and SEM images, as shown in Figure 2. CS including *D*‐glucosamine and *N*‐acetyl‐*D*‐glucosamine was obtained by partial deacetylation of chitin [45]. The characteristic peaks of pure CS are located at approximately 20° because it is a linear semicrystalline polysaccharide (Fig. 2A). The XRD pattern of CS‐SMSC‐126‐Exos is similar to pure CS, because the exosome as an amorphous material does not possess characteristic peaks. Figure 2B shows the FTIR spectra of CS‐SMSC‐126‐Exos and CS hydrogel. For pure CS, the characteristic band due to the stretching vibration of N–H and O–H groups is located at approximately 3,417 cm^−1^. The bands at 2,921 and 2,862 cm^‐1^ correspond to the C–H stretching vibration in the –CH and –CH_2_ groups [46].The band at 1,568 cm^−1^ is assigned to N–H bending vibration overlapping amide II vibration. The bands due to C=O stretching vibration and C–H deformation vibration are located at 1,658 and 1,420 cm^−1^, respectively [47]. The band due to C–O–C stretching vibration modes is observed at 1,030 cm^−1^ [48]. The characteristic bands due to CS were also observed in CS‐SMSC‐126‐Exos (Fig. 2B).

The thermal behaviors of the CS‐SMSC‐126‐Exos and CS hydrogel after freeze‐drying were examined by TG‐DTA curves, as shown in Figure 2C. For both samples, the weight loss of ∼12.3% observed between 25°C and 100°C was ascribed to the loss of physically adsorbed and bound water. The maximum weight loss of ∼57.6% over the temperature range 100–460°C was related to depolymerization of acetylated and deacetylated units of the CS chain. The corresponding exothermic peaks for the CS‐SMSC‐126‐Exos and CS hydrogel were located at 312°C and 293°C, respectively. In addition, the weight loss of ∼30.1% observed over the temperature range 460–580°C was attributed to the decomposition of CS (or exosomes). The corresponding exothermic peaks for the CS‐SMSC‐126‐Exos and CS hydrogel were located at 565°C and 553°C, respectively. Both the CS‐SMSC‐126‐Exos and the CS hydrogel exhibited similar weight loss at all stages. Interestingly, the temperature increases due to exothermic peaks of CS‐SMSC‐126‐Exos were higher than those observed for CS hydrogel, demonstrating further interaction between CS and exosomes.

Figure 2D, 2E shows the morphology of CS‐SMSC‐126‐Exos after freeze‐drying. The low‐resolution SEM image indicates the presence of many macropores with pore sizes of ∼10 μm (Fig. 2D), which originated from the volatilization of water within the CS‐SMSC‐126‐Exos hydrogel during freeze‐drying. Notably, the high‐resolution SEM image shows that CS‐SMSC‐126‐Exos is composed of many nanoparticles (Fig. 2E). These nanoparticles may be aggregates of CS and exosome because of the bonding interactions between them. The corresponding EDS spectrum indicates that the CS‐SMSC‐126‐Exos include both the elements C and O, as well as H (Fig. 2F).

### Controlled Release of SMSC‐126‐Exos

**Figure 3:**
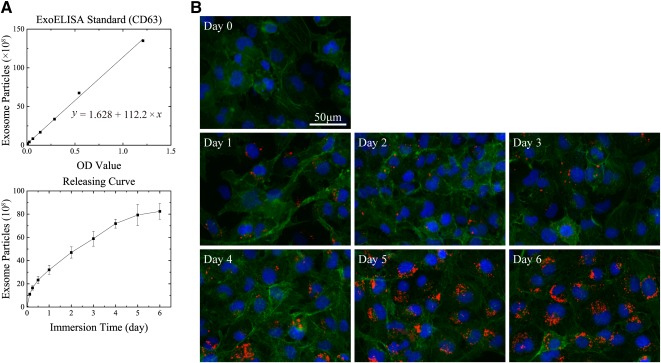
Controlled release of synovium mesenchymal stem cell‐derived exosomes (SMSC‐Exos). **(A):** ExoELISA standard curve and SMSC‐Exos releasing curve. At each time point, three replicates were used. **(B):** Transfection efficiency of supernatant after different immersion times. Three independent experiments were performed to confirm the stability of the phenomenon. Scale bar = 50 μm. Abbreviations: ExoELISA, exosome enzyme‐linked immunosorbent assay kit; OD, optical density.

Immersion of the material in MesenGro hMSC medium for 3, 6, or 12 hours or 1, 2, 3, 4, 5, or 6 days followed by dissolution after 6‐day immersion resulted in an increase in the number of exosome particles detected using an ExoELISA kit. The total load of the hydrogel was 183.08 ± 15.44 × 10^8^ exosome particles. The release curve is shown in Figure 3A.

To detect the release of exosomes from the material, we used a red fluorescent lipophilic dye (Dil) to label SMSCs. The exosomes released by the labeled cells were also labeled with Dil upon fusion of multivesicular bodies with the cell plasma membrane. The labeled exosomes were combined with materials by using the same method as unlabeled exosomes. Cells were cultured with the conditioned medium from each time point for 24 hours. The results showed that the Dil‐labeled exosomes were present in the perinuclear region of HMEC‐1. Furthermore, the number of labeled exosomes in the perinuclear region of HMEC‐1 increased slowly with increased immersion time (Fig. 3B).

### SMSC‐126‐Exos Promote Proliferation of HMEC‐1 and FBs In Vitro

**Figure 4:**
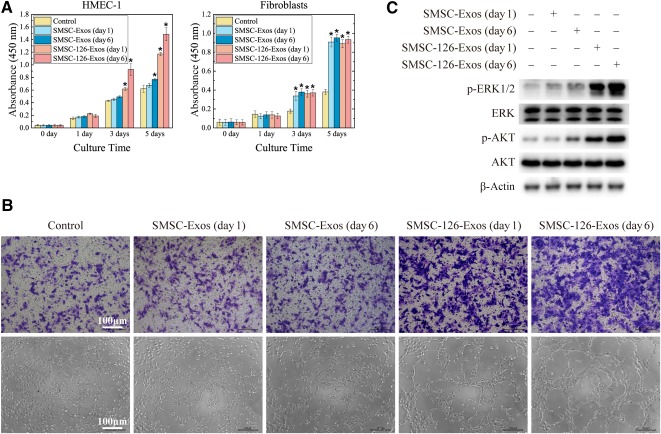
In vitro experiments to evaluate the effects of SMSC‐Exos and SMSC‐126‐Exos. **(A):** Proliferation of HMEC‐1 and fibroblasts incubated for 0, 1, 3, or 5 days in conditioned medium from days 0 and 6. At each time point of each group, three replicates were used. **(B):** Representative photographs showing the effect of conditioned medium from days 0 and 6 on the transwell migration and tubule formation of HMEC‐1. Three independent experiments were performed to confirm the stability of the phenomenon. Scale bars = 100 μm. **(C):** Protein phosphorylation levels of ERK1/2 and AKT analyzed by Western blotting. The experiments were performed three times and a representative image is shown. Abbreviations: ERK, extracellular signal‐regulated kinase; HMEC‐1, human dermal microvascular endothelial cells; p‐ERK1/2, phospho‐extracellular signal‐regulated kinase 1/2; SMSC‐126‐Exos, exosomes derived from microRNA‐126‐3p‐overexpressing synovium mesenchymal stem cells; SMSC‐Exos, synovium mesenchymal stem cell‐derived exosomes.

qRT‐PCR analysis confirmed a significant relative upregulation of miR‐126‐3p in miR‐126‐3p‐overexpressing SMSCs (SMSC‐126) and SMSC‐126‐derived exosomes (SMSC‐126‐Exos) (Fig. 1D). The proliferation of HMEC‐1 and FBs cultured for 1, 3, or 5 days in medium containing SMSC‐Exos or SMSC‐126‐Exos is shown in Figure 4A. Both SMSC‐Exos and SMSC‐126‐Exos increased the proliferation of FBs. SMSC‐126‐Exos obviously increased the proliferation of HMEC‐1, whereas SMSC‐Exos had no significant effect.

Transwell assays showed that SMSC‐126‐Exos significantly promote HMEC‐1 migration compared with SMSC‐Exos or control (Fig. 4B). Tube formation assays using HMEC‐1 were used to determine the proangiogenic potential of the SMSC‐126‐Exos. After incubating on Matrigel substratum for 6 hours, HMEC‐1 incubated with control medium and SMSC‐Exos formed sparse or even incomplete tube networks, whereas SMSC‐126‐Exos significantly enhanced the tube‐forming ability of HMEC‐1 (Fig. 4B).

Western blotting showed that SMSC‐126‐Exos significantly activated AKT and ERK1/2 (Fig. 4C). The results showed that SMSC‐126‐Exos could have great potential for improved wound healing.

### SMSC‐126‐Exos Promote Cutaneous Wound Healing in a Diabetic Rat Model

**Figure 5:**
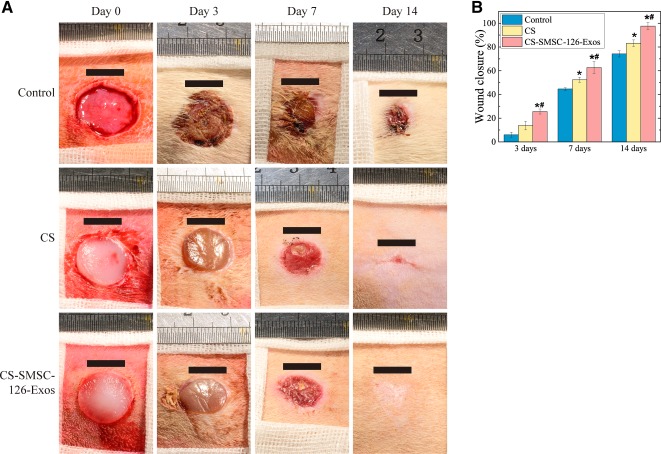
Wound closures were significantly promoted by CS‐SMSC‐126‐Exos. **(A):** Representative images of full‐thickness skin defects, left untreated (control) or treated with CS or miR‐126‐3p‐overexpressing synovium mesenchymal stem cells combined with CS, at 0, 3, 7, and 14 days after surgery. Scale bars = 10 mm. **(B):** Percent wound closure. ∗, *p* < .05 compared with control; #, *p* < .05 compared with CS. Six wounds per group at each time point were used for statistical analysis. Abbreviations: CS, chitosan; CS‐SMSC‐126‐Exos, exosomes derived from microRNA‐126‐3p‐overexpressing synovium mesenchymal stem cells combined with chitosan.

No death or abnormality was observed in any animal during the postoperative period. Figure 5A shows general optical images of an untreated defect (control), a defect treated with CS hydrogel alone (CS), and a defect treated with CS hydrogel loaded with SMSC‐126‐Exos (CS‐SMSC‐126‐Exos). Although the wounds in all three groups reduced in size, the wound sizes in the groups treated with CS and CS‐SMSC‐126‐Exos were smaller than in the untreated control group. In particular, wounds treated with CS‐SMSC‐126‐Exos had closed by day 14, and wounds treated with CS had almost closed by day 14, whereas the untreated wounds had not.

Quantitative data on wound closure confirmed that wounds treated with CS‐SMSC‐126‐Exos closed significantly faster than untreated wounds at all three treatment times (days 3, 7, and 14) (Fig. 5B). The wounds treated with CS alone showed significantly better healing at day 14 compared with the untreated wounds, but treatment with CS‐SMSC‐126‐Exos provided better wound closure than either controls or CS.

### μCT Evaluation of Wound Healing

**Figure 6:**
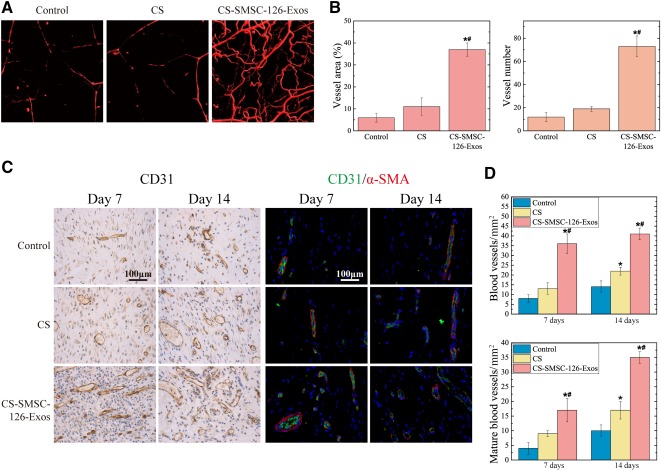
In vivo experiments to observe the promoted vascularization. **(A):** μCT images of blood vessel formation in full‐thickness skin defects left untreated (control) or treated with CS or exosomes derived from miR‐126‐3p‐overexpressing synovium mesenchymal stem cells (SMSC‐126‐Exos) combined with CS at 14 days after surgery. **(B):** Morphometric analysis of the new blood vessel area and the number of blood vessels. ∗, *p* < .05 compared with control; #, *p* < .05 compared with CS. Three wounds per group were used for statistical analysis. **(C):** Immunohistochemical staining of CD31 (left) and immunofluorescent staining of CD31 and α‐SMA (right). New vessels were defined by positive CD31 staining and their typical oval structure. Endothelial cells (CD31), smooth muscle cells (α‐SMA), and cell nuclei are stained green, red, and blue, respectively, at 7 and 14 days after surgery. ∗, *p* < .05 compared with control; #, *p* < .05 compared with CS. Three wounds per group at each time‐point were used for statistical analysis. Scale bars = 100 μm. **(D):** Statistical results from **C**. ∗, *p* < .05 compared with control; #, *p* < .05 compared with CS. Abbreviations: CS, chitosan; CS‐SMSC‐126‐Exos, exosomes derived from microRNA‐126‐3p‐overexpressing synovium mesenchymal stem cells combined with chitosan; α‐SMA, α‐smooth muscle actin.

After day 14, the formation of blood vessels in untreated defects and in defects treated with CS or CS‐SMSC‐126‐Exos was evaluated by using μCT. The three‐dimensional reconstructed images (Fig. 6A) showed a much higher number of blood vessels in defects treated with CS‐SMSC‐126‐Exos than in defects treated with CS alone or in untreated defects. Quantitative analysis of the newly formed blood vessels showed a significantly higher blood vessel number and blood vessel area in the defects treated with CS‐SMSC‐126‐Exos than in the untreated defects or those treated with CS alone (Fig. 6B).

### Histologic, Immunohistochemical, and Immunofluorescent Analysis

**Figure 7:**
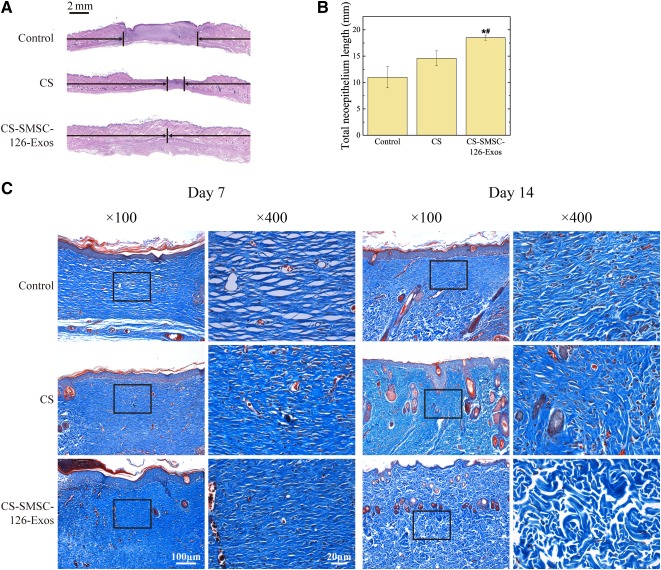
In vivo experiments to observe the promoted re‐epithelialization. **(A):** Transmitted light images of H&E‐stained sections of untreated defects (control) and defects treated with CS or miR‐126‐3p‐overexpressing synovium mesenchymal stem cells (SMSC‐126‐Exos) combined with CS at 14 days after surgery. Scale bar = 2 mm. **(B):** Total neoepithelium length in the skin defects left untreated (control) or treated with CS or SMSC‐126‐Exos combined with CS at 14 days after surgery. ∗, *p* < .05; compared with control; #, *p* < .05 compared with CS. Three wounds per group were used for statistical analysis. **(C):** Transmitted light images of a Masson’s trichrome‐stained section of an untreated (control) defect and a defect treated with CS or SMSC‐126‐Exos combined with CS at 7 and 14 days after surgery, showing collagen deposition. At each time point of each group, three replicates were used, and representative images are shown. Scale bars = 100 μm (×100) and 20 μm (×400). Abbreviations: CS, chitosan; CS‐SMSC‐126‐Exos, exosomes derived from microRNA‐126‐3p‐overexpressing synovium mesenchymal stem cells combined with chitosan.

As shown in Figure 7A, 7B, CS‐SMSC‐126‐Exos significantly enhanced re‐epithelialization, compared with that observed in control wounds and those treated with CS without exosomes. Masson’s trichrome staining showed almost complete re‐epithelialization, thicker and more mature granulation tissue, and a large improvement in collagen alignment and deposition in the defects treated with CS‐SMSC‐126‐Exos compared with defects treated with CS alone or untreated control, which indicated the positive influence of exosomes on extracellular matrix (ECM) remodeling (Fig. 7C). Masson’s trichrome staining showed that SMSC‐126‐Exos accelerated the development of hair follicles and sebaceous glands at 14 days after surgery.

Staining for CD31, a transmembrane protein expressed in early vascular development, was used to identify newly formed vessels. The formation of mature vessels was characterized by costaining of CD31 and α‐SMA, from which average vessel densities and the number of mature vessels was quantified (Fig. 6C, 6D). The number of both newly formed vessels and mature vessels increased during the wound‐healing process. The CS‐SMSC‐126‐Exos group showed the highest vessel numbers and densities of mature vessels at days 7 and 14, compared with the CS alone and control groups.

## Discussion

Slow angiogenesis can result in cell death in an ischemic region, which hinders wound recovery and tissue regeneration, especially when associated with diabetes [49]. Hence, rapid angiogenesis is extremely important for effective tissue reconstruction. Among the strategies for enhancing angiogenesis, the use of exosomes has attracted more and more attention recently [50, 51].

Several reports have shown a beneficial effect of CS as a biologically active dressing in wound healing. CS hydrogel wound dressings are hemostatic, antibacterial, biodegradable, and biocompatible [34]. It has been reported that the appliction of CS to open wounds results in high growth factor activity, increased infiltration by inflammatory cells, and increased formation of granulation tissue, along with angiogenesis [52, 53, 54]. In addition, CS hydrogel wound dressings are a good carrier for sustained‐release materials such as nanoparticles [33] or, in our study, exosomes.

We found that SMSC‐Exos significantly promote fibroblast proliferation, but have an important drawback in that SMSC‐Exos cannot strongly promote angiogenesis. Hence, we modified the SMSCs using gene‐overexpression technology and successfully overexpressed miR‐126‐3p. Through RT‐qPCR analysis, we were delighted to find that miR‐126‐3p was significantly increased in SMSC‐126‐Exos compared with SMSC‐Exos. This modification of SMSCs also rendered their exosomes (SMSC‐126‐Exos) as more powerful in inactivating angiogenesis. Recent studies have revealed the necessary role of miR‐126 in EPCs, which are important in neoangiogenesis and tissue repair in ischemic diseases [13]. We hypothesized that overexpression of miR‐126‐3p would confer this function of EPCs on SMSCs. Isolation and modification of SMSCs is faster and more cost‐effective compared with isolation and cultivation of EPCs.

We found that SMSC‐126‐Exos were able to significantly activate the PI3K/AKT and MAPK/ERK pathways compared with SMSC‐Exos. These additional functions of SMSC‐126‐Exos are very similar to the function of miR‐126 [15, 16]. Both AKT and ERK1/2 activation play key roles in cell proliferation, migration, and angiogenesis [55]. Modification through gene overexpression can improve the function of exosomes or even confer the function of rare cell types on more easily obtained cells. This kind of approach promises huge future returns.

The abilities of SMSC‐Exos to promote fibroblast proliferation were inherited by the SMSC‐126‐Exos. Fibroblasts are connective tissue cells required for the repair of tissue injury and are responsible for collagen deposition. Because collagen is the main component of the ECM and is largely responsible for its support and strength [56], this provides a beneficial cradle for wound healing.

In previous studies, the gelatinization of CS was usually performed at high temperature such as 76°C in a boiling water bath [57], 60°C for 24 hours [58], or 80°C for 3.5 hours [59]. Indeed, such high‐temperature polymerization is the easiest method of obtaining regular CS hydrogel. However, high temperatures are harmful to exosomes. In our study, we therefore attempted to polymerize CS at a low temperature to avoid deleterious effects on the normal function of exosomes. After several attempts, we found a solution to this problem. Before polymerization, the mixture was placed at −20°C for 2 hours, then polymerized following the method described in this article. We found that the low‐temperature‐polymerized CS hydrogel showed no obvious differences in microstructure or macrostructure compared with high‐temperature‐polymerized CS hydrogel. Previous studies have shown that the main underlying mechanism of stem cell transplantation therapy is likely to depend on the paracrine effects of stem cells, and the administration of exosomes is sufficient to trigger the repair process in various disease models.

In consideration of cost, chitosan alone, which is cheaper, could promote wound closure, but lacked the angiogenic ability provided by SMSC‐126‐Exos, and the recovery below the surface of the skin was not as good (Fig. 7A). In fact, obtaining and culturing SMSCs is really inexpensive, requiring no special or expensive additional agents. Because the doubling time of SMSCs is approximately 2 days, expanding the cells in culture does not take too much time. At first, the design, construction and verification of the overexpressing lentivirus would incur some costs, but the subsequent plasmid amplification and lentivirus packaging would be relatively inexpensive. In the future, with the help of new technology, including hollow fiber bioreactors [60], which have the potential to greatly enhance the harvest rate of exosomes, the consumption of exosomes would be less and less. Therefore, exosome‐based therapy could become a promising mainstream future therapy strategy.

Our results clearly demonstrate that the controlled release of SMSC‐126‐Exos greatly increases re‐epithelization and collagen deposition at wound sites. SMSC‐126‐Exos not only activate the generation of newly formed vessels, but also accelerate their maturation.

## Conclusion

In this study, we demonstrate that CS can provide controlled release of SMSC‐126‐Exos and thus have beneficial effects on wound healing by increasing the formation of granulation tissue and angiogenesis, which are the two major phases of cutaneous wound healing. Our findings suggest that a material with controlled‐release function combined with exosomes derived from a modified cell line could be a promising novel therapy for wound healing.

## Author Contributions

S.‐C.T.: collection and/or assembly of data, data analysis and interpretation, manuscript writing; S.‐C.G.: administrative support, financial support; M.L.: provision of study materials; Q.‐F.K.: collection and/or assembly of data; Y.‐P.G.: data analysis and interpretation, financial support; C.‐Q.Z.: conception and design, final approval of manuscript.

## Disclosure of Potential Conflicts of Interest

The authors indicated no potential conflicts of interest.
